# Disability: The Understudied Population in Orthopaedics

**DOI:** 10.7759/cureus.103842

**Published:** 2026-02-18

**Authors:** Ally A Yang, Meera M Dhodapkar, Julian Smith-Voudouris, Corinna C Franklin, David B Frumberg

**Affiliations:** 1 Department of Orthopaedics and Rehabilitation, Yale University, New Haven, USA; 2 Department of Orthopaedic Surgery, Mayo Clinic, Rochester, USA

**Keywords:** disability, healthcare disparity, health equity, orthopedic surgery, systematic review, underserved populations

## Abstract

Healthcare disparities profoundly affect healthcare delivery. Patients with disabilities remain under-recognized as a population warranting focused public health attention. The purpose of this systematic review was to identify and characterize published literature addressing access to or outcomes of orthopaedic care among people with disabilities.

This study received IRB exemption. Articles published within the past five years in the top 20 highest impact factor (IF), Medline-indexed American orthopaedic surgery journals were identified through PubMed. Studies assessing access to or outcomes of orthopaedic care among underserved populations were included. Two reviewers independently screened abstracts and full-text articles and extracted data, with discrepancies resolved by consensus.

A total of 124 manuscripts met the inclusion criteria. Of these, 61 (49.2%) only examined disparities in access, 36 (29.0%) examined disparities in outcomes, and 27 (21.8%) examined disparities in both access and outcomes. Commonly evaluated disparity-related factors were race (53.2% of outcomes studies and 32.6% of access studies), insurance/payor status (29.0% outcomes and 39.3% access), socioeconomic factors (45.2% outcomes and 28.1% access), ethnicity (33.9% outcomes and 19.1% access), and presence of chronic illnesses (35.5% outcomes and 30.3% access). Barriers to orthopaedic care access or orthopaedic outcomes related to disability were examined less often than those related to other underserved populations, in only 6 (5.0%) of all studies.

There remains a critical gap in the orthopaedic literature regarding access to care and outcome disparities among patients with disabilities. Future research should systematically evaluate barriers faced by this population and incorporate inclusive design principles to promote equitable orthopaedic care.

## Introduction and background

Health disparities in orthopaedic surgery

Healthcare is profoundly influenced by race, ethnicity, sex, and socioeconomic status, all of which contribute significantly to healthcare inequities. Members of marginalized and underserved populations [1] have been disproportionately affected and remain underrepresented in health research. As disparities in musculoskeletal care have received growing attention [2], there has been increasing research in both the general medical literature and the orthopaedic surgery literature assessing access to care and outcomes among historically marginalized groups [3-6]. Common factors examined include race, ethnicity, living situation, employment, income, insurance status, education, and social support.

Disability as an understudied determinant of orthopaedic care

However, disability, defined by the Americans with Disabilities Act (1990) as “a physical or mental impairment that substantially limits one or more major life activities,” remains an under-studied determinant of health within orthopaedics [7]. Over 61 million Americans, nearly 30% of the adult US population [8], have disabilities, and mounting evidence indicates that they experience healthcare disparities compared to individuals without disabilities [3,4,9-11]. This study focuses on physical disabilities and intellectual limitations.

Despite having greater healthcare needs, patients with disabilities often face reduced access to essential services, including cancer screenings, oral healthcare, and cholesterol checks. Additional barriers include physical inaccessibility of healthcare facilities and examination rooms, communication difficulties with providers, and potential bias or lack of understanding from healthcare professionals [3,5,11-14].

In contrast to fields such as oncology and rehabilitation medicine [15,16], where disparity frameworks and accessibility initiatives are increasingly emphasized [17], orthopaedic research has lagged in evaluating how disability affects access to and outcomes of musculoskeletal care. Prior orthopaedic disparity studies have largely focused on race, ethnicity, or socioeconomic status [18,19], leaving disability as a largely unexamined domain.

The purpose of this systematic review is to identify and characterize published literature on barriers to orthopaedic care and disparities in outcomes among patients with disabilities in US orthopaedic surgery journals. We hypothesized that disability is markedly underrepresented relative to other underserved populations.

## Review

Methods

The systematic review was designed and reported in accordance with the Strengthening the Reporting of Observational Studies in Epidemiology (STROBE) and the Preferred Reporting Items for Systematic Reviews and Meta-Analyses (PRISMA) guidelines. This study received exemption from IRB oversight.

Eligibility Criteria

Articles examining disparities in access to or outcomes of orthopaedic care, published in the top 20 highest impact-factor (IF), Medline-indexed American orthopaedic surgery journals within the past five years, were queried. Only studies reporting on US patient populations were included in the final analysis.

Information Sources and Search Strategy

The search strategy (Appendix 1) and study inclusion criteria were developed with the assistance of a qualified medical librarian. Primary search terms included “access” and “underserved” to broadly capture disparity-related literature. These terms were applied in combination with journal-specific filters, restricting results to the top 20 highest IF, Medline-indexed American orthopaedic surgery journals. Disability-related disparities were identified during title, abstract, and full-text screening. Journal characteristics and subject matter were confirmed using Clarivate Journal Citation Reports, and the five-year journal IF was used to identify the top 20 journals. The search was intentionally limited to US-based journals to align findings with the Americans with Disabilities Act (ADA) framework; however, this may introduce publication, language, and geographic bias, which are further acknowledged in the Limitations. A comprehensive search was conducted in PubMed on March 14, 2023. 

Study Selection

Article selection and data extraction were conducted using Covidence (Melbourne, Australia), a systematic review software program. Two rounds of screening were performed to determine article eligibility: first, title and abstract screening, followed by full-text review. Two researchers (A.Y. and M.D.) independently and in duplicate evaluated the titles and abstracts of all potentially eligible articles. Remaining articles from this pool underwent full-text screening, also conducted in duplicate to determine final eligibility. The reference lists of included studies were reviewed to identify additional citations for full-text screening. Any conflicts regarding article inclusion were resolved by consensus among the reviewers, including a senior author (A.Y., M.D., D.F.).

Data Extraction and Variables

**Figure 1:**
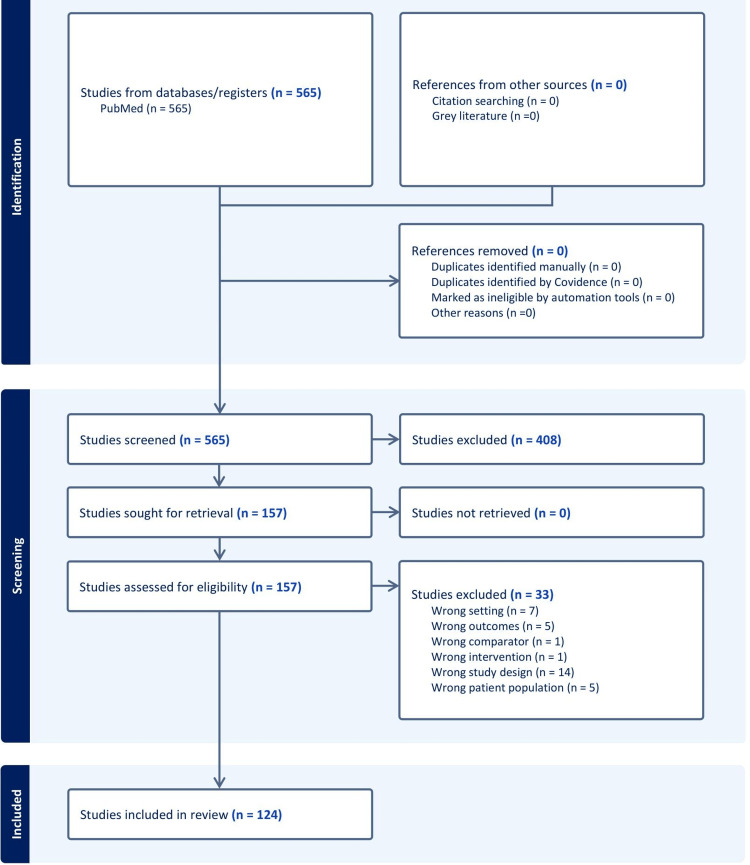
PRISMA flowchart of the study identification process

Data extraction was performed in duplicate (A.Y., M.D.) to ensure accuracy. Extracted information included the orthopaedic subspecialty addressed in each manuscript, whether disparities in outcomes, access, or both were assessed, manuscript type (e.g., original research article/letter, systematic review/meta-analysis, commentary/letter to the editor), study design (for original research articles), whether disability was mentioned, and, if so, in what capacity (e.g., primary outcome/focus, secondary outcome/sub-analysis). Additional marginalized groups assessed were also recorded, including racial and ethnic minorities, gender disparities, sexual orientation, socioeconomic factors, insurance/payor status, and medical comorbidities such as chronic illnesses. Study titles, abstracts, and full texts were examined for explicit mention or evaluation of disability. Studies were classified as addressing disability if they evaluated populations with diagnosed physical disabilities (e.g., tetraplegia), intellectual or developmental disabilities, functional impairments, or if disability was operationalized using proxy measures such as ambulatory status or composite indices incorporating disability-related variables. Disability classification was based on how individual studies defined and measured disability. Figure 1 outlines the study identification, screening, and inclusion.

Risk-of-Bias Assessment

A risk of bias assessment was not performed, as the objective of this systematic review was to characterize the representation of disability in orthopaedic disparity literature rather than to evaluate comparative clinical outcomes.

Results

Study Characteristics

The literature search yielded a total of 565 citations. After removal of duplicates, 565 unique citations remained. Following title and abstract screening, 157 articles underwent full-text review, and 124 articles were included in the final data extraction (Figure 1).

Among the 124 included manuscripts, 61 (49.2%) examined disparities in access to orthopaedic care, 36 (29.0%) examined disparities in outcomes, and 27 (21.8%) examined both access and outcomes. The majority were original research articles, with the remainder comprising commentaries, reviews, and systematic reviews. By subspecialty, the largest proportion focused on arthroplasty (44.4%), followed by general orthopaedics (10.5%) and spine (9.7%) (Table 1). Smaller proportions addressed pediatrics, shoulder and elbow, trauma, sports, and oncology (Table 1).

**Table 1:** Articles included in the study and specialties of focus

	Articles
Total	124
Orthopaedic subspecialty	
Joints	55 (44.4%)
Hand	2 (1.6%)
Orthopaedic oncology	6 (4.8%)
General orthopaedics	13 (10.5%)
Pediatrics	10 (8.1%)
Shoulder and elbow	10 (8.1%)
Spine	12 (9.7%)
Sports	6 (4.8%)
Trauma	9 (7.3%)
Other	1 (0.8%)
Manuscript type	
Commentary/letter to the editor	30 (24.2%)
Original research article/research letter	84 (67.7%)
Review article	7 (5.6%)
Systematic review/meta-analysis	3 (2.4%)

Disparities Examined Across Studies

Patient characteristics most commonly examined in relation to disparities in outcomes or access to care were, in descending order: race (53.2% of outcomes studies and 32.6% of access studies), insurance/payor status (29.0% outcomes and 39.3% access), socioeconomic factors (45.2% outcomes and 28.1% access), ethnicity (33.9% outcomes and 19.1% access), and presence of chronic illnesses (35.5% outcomes and 30.3% access). In contrast, sex, language-related factors, and gender identity or sexual orientation were examined less frequently (Table 2).

**Table 2:** Access and outcomes articles included in the study and topics of focus for each

	Outcomes studies, n (%)	Access studies, n (%)
Topics of focus		
Disability	4 (6.5%)	2 (2.2%)
Insurance/payor	18 (29.0%)	35 (39.3%)
Race	33 (53.2%)	29 (32.6%)
Ethnicity	21 (33.9%)	17 (19.1%)
Socioeconomic, non-insurance, health literacy, and geographic	28 (45.2%)	25 (28.1%)
Chronic illnesses and age	22 (35.5%)	27 (30.3%)
Language (e.g., non-primary English speakers)	2 (3.2%)	5 (5.6%)
Sex	13 (21.0%)	11 (12.4%)
Sexual orientation or gender identity	1 (1.6%)	1 (1.1%)

Disability-Focused Studies

Barriers to orthopaedic care access or outcomes related to disability were examined far less frequently than those related to other underserved populations, appearing in only 5.0% (n = 6) of all included studies (Table 3). Among studies mentioning disability, 2 (33.3%) evaluated barriers to access, while 4 (44%) assessed outcomes for patients with disabilities. Four studies (66.7%) analysed disability as a sub-analysis or secondary outcome (Table 3). These studies spanned multiple orthopaedic subspecialties and were predominantly retrospective original research (Table 3). A complete list of all included studies is provided in Table 4.

**Table 3:** Summary of studies addressing disability-related disparities in orthopaedic care

Study (author, year)	Subspecialty	Study design	Role of disability in study	Type of disparity assessed	Disability-related findings
Liljenquist et al. (2018) [20]	General orthopaedics	Retrospective cohort	Primary outcome	Access	Frequency of physical therapy services decreases once young adults with cerebral palsy leave secondary school
Huynh et al. (2021) [21]	Hand	Retrospective cohort	Primary outcome	Access	Rate of upper extremity reconstruction for patients with tetraplegia is low and is correlated with the environment of care, financial factors, and provider availability
Holbert et al. (2022) [22]	Arthroplasty	Retrospective cohort	Secondary analysis	Outcome	Patients with disabilities were less likely to be discharged home and demonstrated higher 90-day postoperative ED utilization, indicating more frequent postoperative complications
Fourman et al. (2023) [23]	Orthopaedic oncology	Commentary	Secondary analysis	Outcome	Preoperative non-ambulatory status was associated with substantially poorer postoperative mobility and functional recovery
Engler et al. (2023) [24]	Orthopaedic oncology	Retrospective cohort	Secondary analysis	Outcome	Disability-related components of community-level social vulnerability contributed to disparities in postoperative outcomes following spine surgery
De la Garza Ramos et al. (2023) [25]	Spine surgery	Retrospective cohort	Secondary analysis	Outcome	Preoperative non-ambulatory status was the strongest independent predictor of postoperative ambulatory limitations, even after adjusting for race, social vulnerability, and clinical factors

Discussion

The current study aimed to identify and characterize published literature from the past five years in the top 20 highest IF American orthopaedic journals, focusing on barriers to orthopaedic care and disparities in outcomes among patients with disabilities in the United States. Of 124 included articles, only 6 (5.0%) examined barriers to access or disparities in outcomes for patients with disabilities. Moreover, only about one-third of these studies assessed disability as a primary outcome, while the majority (66%) considered disability as a secondary outcome or sub-analysis.

These findings highlight a striking underrepresentation of disability-focused research compared with other disparity domains in orthopaedics. Few studies have examined the health disparities experienced by individuals with disabilities [1,9,11,13,14], despite evidence that these patients report higher rates of physical inactivity, inadequate emotional support, and worse self-reported health status compared with patients without disabilities [3,4,9-11]. Patients with disabilities also have a higher prevalence of chronic conditions, including cardiac disease, hypertension, hyperlipidaemia, diabetes, stroke, arthritis, and asthma [26]. Many healthcare barriers faced by patients with disabilities overlap with those experienced by other underserved populations, such as limited access to appointments [4], lack of accessible or timely transportation [27], financial and insurance-related obstacles [6], poor physician-patient communication [28], and discrimination [9,10]. In addition, patients with disabilities may encounter unique barriers, including insufficient insurance coverage and implicit bias or unconscious negative attitudes from healthcare providers [1].

Implications for Practice and Research

Several structural and systemic factors likely contribute to the underrepresentation of disability-focused research in the orthopaedic literature. Much orthopaedic disparity research relies on administrative claims and registry datasets, which often lack standardized measures of functional status or disability. As a result, proxy variables such as ambulatory status must be used, potentially introducing misclassification [29]. Additionally, disability-related information is not as consistently recorded in predefined fields of electronic medical records as other commonly studied disparity characteristics, such as race, ethnicity, or gender [30]. Finally, disability has traditionally been conceptualized as a comorbidity rather than a distinct disparity domain, leading to disability variables being adjusted for in analyses rather than investigated as the primary exposure [31].

According to the US Bone and Joint Initiative, musculoskeletal diseases account for more than 50% of disabling health conditions reported by adults [26]. In a study by Zaidel et al., short-term disability claims were most commonly associated with musculoskeletal disorders (23%), followed by musculoskeletal injuries (17%) [32]. Understanding the unique barriers to quality orthopaedic care experienced by people with disabilities is essential for developing interventions to improve access and outcomes for this historically marginalized group. Our study highlights substantial gaps in the literature, emphasizing the need for future research in this area.

In addition to the limited literature on disability-related disparities, we found that among the 124 manuscripts meeting inclusion criteria (Figure 1), only 1 (0.8%) examined disparities related to gender identity or sexual orientation. Gender and sexual minority patients have historically faced disparities in access to care and health outcomes [33], which are well documented in other healthcare settings. Our findings indicate that research examining the unique challenges these patients face in accessing equitable orthopaedic care is extremely limited, representing an important area for future study in the field.

Limitations

This study has several limitations. First, our focus was limited to a subset of orthopaedic journals published over the past five years. While restricting the analysis to the top 20 highest IF journals allows investigation of potentially higher-impact literature, this approach may have excluded relevant studies published in lower-impact journals, reflecting a trade-off between selectivity and completeness. Nevertheless, we believe the study provides valuable insight into the overall landscape of orthopaedic literature on this topic. Second, we only examined American journals, given the unique impact of the ADA on healthcare and the potential limited applicability of international studies to orthopaedic care in the United States. Additionally, disability was not uniformly defined across all included studies, which may introduce misclassification bias and limit the accuracy of disability-related findings. Despite these limitations, our study offers important insight into gaps in the literature and highlights areas for future research in the orthopaedic community. 

## Conclusions

There is a critical gap in the orthopaedic surgery literature regarding access to care for patients with disabilities, underscoring the need for their systematic inclusion in future research. Addressing this omission is essential. Future studies should incorporate disability-related variables into national orthopaedic datasets, ensure accessible design in surgical and rehabilitation environments, and prioritize evaluation of barriers to care for patients with physical or cognitive impairments. By recognizing disability as a central domain of health disparity alongside race, gender, and socioeconomic status, orthopaedics can better align with the accessibility principles of the ADA and provide equitable care to all patients.

## Appendices

Appendix 1

Search Strategy to Identify Study Articles

(("access") OR ("underserved")) AND ("0363-5465"[Journal] OR "0021-9355"[Journal] OR "0190-6011"[Journal] OR "0009-921X"[Journal] OR "0749-8063"[Journal] OR "0883-5403"[Journal] OR "1935-973X"[Journal] OR "1935-973X"[Journal] OR "1529-9430"[Journal] OR "1529-9430"[Journal] OR "0031-9023"[Journal] OR " 1067-151X"[Journal] OR " 2572-1143"[Journal] OR " 1058-2746"[Journal] OR " 1071-1007"[Journal] OR " 0362-2436"[Journal] OR " 0736-0266"[Journal] OR " 1947-6035"[Journal] OR " 0890-5339"[Journal] OR " 0030-5898"[Journal] OR “0363-5023” [Journal] OR “0271-6798”[Journal])

Appendix 2

**Table 4:** Summary of the included studies in this systematic review

Study (author, year)	Subspecialty	Disability mentioned	Type of disparity assessed
Liljenquist et al. (2018) [20]	Pediatrics	Yes	Access
Holbert et al. (2022) [22]	Joints	Yes	Outcomes
Fourman (2023) [23]	Orthopaedic oncology	Yes	Outcomes
Engler et al. (2023) [24]	Spine	Yes	Outcomes
De la Garza Ramos et al. (2023) [25]	Orthopaedic oncology	Yes	Outcomes
Huynh et al. (2021) [21]	Hand	Yes	Access
Zuckerman (2021) [34]	Other: general orthopaedics	No	Access
Yousman et al. (2021) [35]	Other: general orthopaedics	No	Access
Yatham et al. (2023) [36]	Orthopaedic oncology	No	Access
Xiong et al. (2021) [37]	Other: general orthopaedics	No	Access
Wiznia et al. (2020) [38]	Other: general orthopaedics	No	Access
Wiznia et al. (2022) [39]	Joints	No	Access
Wiznia et al. (2022) [40]	Joints	No	Access
Wiznia et al. (2022) [41]	Joints	No	Access
Wiznia et al. (2022) [42]	Joints	No	Access
Wiznia et al. (2022) [43]	Joints	No	Access
Williamson et al. (2020) [44]	Other: general orthopaedics	No	Access
Wick et al. (2022) [45]	Spine	No	Access
Wang et al. (2022) [46]	Spine	No	Access
Wang et al. (2018) [47]	Joints	No	Access
Tartarilla et al. (2022) [48]	Pediatrics	No	Access
Suchman et al. (2018) [49]	Sports	No	Access
Springer (2019) [50]	Joints	No	Access
Sobel et al. (2020) [51]	Pediatrics	No	Access
Shaw (2018) [52]	Joints	No	Access
Schwartz et al. (2019) [53]	Joints	No	Access
Schottel and Nichols (2018) [54]	Trauma	No	Access
Sabesan et al. (2022) [55]	Joints	No	Access
Sabesan et al. (2022) [56]	Joints	No	Access
Ries (2023) [57]	Joints	No	Access
Ratnasamy et al. (2023) [58]	Joints	No	Access
Ramirez et al. (2022) [59]	Joints	No	Access
Rahman et al. (202)1 [60]	Joints	No	Access
Rabah et al. (2020) [61]	Other: general orthopaedics	No	Access
Potak and Iobst (2019) [62]	Pediatrics	No	Access
O'Connor et al. (2022) [63]	Joints	No	Access
O'Connor et al. (2022) [64]	Joints	No	Access
O'Connor et al. (2022) [65]	Joints	No	Access
Nguyen et al. (2023) [66]	Joints	No	Access
Nezwek et al. (2021) [67]	Spine	No	Access
Meislin (2022) [68]	Shoulder and elbow	No	Access
McIntyre (2021) [69]	Shoulder and elbow	No	Access
Manner (2022) [70]	Joints	No	Access
Malik et al. (2021) [71]	Orthopaedic oncology	No	Access
Malik et al. (2021) [72]	Trauma	No	Access
Lin et al. (2022) [73]	Joints	No	Access
Jones et al. (2022) [74]	Joints	No	Access
Ibrahim et al. (2022) [75]	Trauma	No	Access
Huff et al. (2022) [76]	Joints	No	Access
Heffernan et al. (2022) [77]	Pediatrics	No	Access
Hartnett et al. (2022) [78]	Joints	No	Access
Gregory et al. (2021) [79]	Shoulder and elbow	No	Access
Greene et al. (2019) [80]	Joints	No	Access
Glassman et al. (2019) [81]	Spine	No	Access
Dy et al. (2019) [82]	Joints	No	Access
Dy et al. (2019) [83]	Other: general orthopaedics	No	Access
Dy et al. (2020) [84]	Joints	No	Access
Durand et al. (2022) [85]	Spine	No	Access
Dlott et al. (2023) [86]	Joints	No	Access
Congiusta et al. (2019) [87]	Trauma	No	Access
Bertrand (2022) [88]	Orthopaedic oncology	No	Access
Beck et al. (2020) [89]	Sports	No	Access
Beck et al. (2019) [90]	Trauma	No	Access
Ayoade et al. (2020) [91]	Hand	No	Access
Arant et al. (2022) [92]	Pediatrics	No	Access
Amen et al. (2022) [93]	Spine	No	Access
Amen et al. (2022) [94]	Joints	No	Access
Almaguer et al. (2019) [95]	Joints	No	Access
Zhuang et al. (2021) [96]	Spine	No	Outcomes
Stroud et al. (2022) [97]	Orthopaedic oncology	No	Outcomes
Shaw et al. (2021) [98]	Joints	No	Outcomes
Sequeira et al. (2023) [99]	Shoulder and elbow	No	Outcomes
Saks et al. (2021) [100]	Sports	No	Outcomes
Rubenstein et al. (2020) [101]	Joints	No	Outcomes
Okike et al. (2022) [102]	Joints	No	Outcomes
Okike et al. (2018) [103]	Trauma	No	Outcomes
Okike et al. (2019) [104]	Joints	No	Outcomes
Moverman et al. (2022) [105]	Shoulder and elbow	No	Outcomes
Mohanty et al. (2022) [106]	Spine	No	Outcomes
Mohanty et al. (2022) [107]	Spine	No	Outcomes
Manner et al. (2021) [108]	Trauma	No	Outcomes
Mandalia et al. (2023) [109]	Shoulder and elbow	No	Outcomes
MacMahon et al. (2023) [110]	Joints	No	Outcomes
Luster et al. (2023) [111]	Pediatrics	No	Outcomes
Kurucan et al. (2020) [112]	Trauma	No	Outcomes
Koressel et al. (2022) [113]	Joints	No	Outcomes
Koressel et al. (2022) [114]	Joints	No	Outcomes
Klemt et al. (2021) [115]	Joints	No	Outcomes
Khlopas et al. (2022) [116]	Joints	No	Outcomes
Kamath et al. (2022) [117]	Joints	No	Outcomes
Hinman et al. (2020) [118]	Joints	No	Outcomes
Gabriel (2020) [119]	Pediatrics	No	Outcomes
Dawes et al. (2023) [120]	Shoulder and elbow	No	Outcomes
Chisari et al. (2021) [121]	Joints	No	Outcomes
Adelani et al. (2023) [122]	Joints	No	Outcomes
Ziedas et al. (2022) [123]	Sports	No	Outcomes; access
Zelle et al. (2021) [124]	Trauma	No	Outcomes; access
Young et al. (2020) [125]	Pediatrics	No	Outcomes; access
Wright et al. (2023) [126]	Other: general orthopaedics	No	Outcomes; access
Vega et al. (2023) [127]	Joints	No	Outcomes; access
Tan et al. (2019) [128]	Joints	No	Outcomes; access
Suleiman et al. (2022) [129]	Joints	No	Outcomes; access
Shau et al. (2018) [130]	Joints	No	Outcomes; access
Salazar et al. (2019) [18]	Other: general orthopaedics	No	Outcomes; access
Rubenstein et al. (2019) [131]	Sports	No	Outcomes; access
Roth et al. (2021) [132]	Joints	No	Outcomes; access
Reyes et al. (2023) [133]	Spine	No	Outcomes; access
Ramsey et al. (2021) [134]	Other	No	Outcomes; access
Ponnusamy et al. (2018) [135]	Joints	No	Outcomes; access
Ottesen et al. (2022) [136]	Other: general orthopaedics	No	Outcomes; access
O'Connor (2020) [137]	Other: general orthopaedics	No	Outcomes; access
Navarro et al. (2019) [138]	Sports	No	Outcomes; access
Mehta et al. (2022) [139]	Joints	No	Outcomes; access
Karimi et al. (2023) [140]	Joints	No	Outcomes; access
Handcox et al. (2022) [141]	Other: general orthopaedics	No	Outcomes; access
Emara et al. (2022) [142]	Joints	No	Outcomes; access
Chun et al. (2021) [143]	Joints	No	Outcomes; access
Cheah et al. (2020) [144]	Joints	No	Outcomes; access
Bridges et al. (2023) [145]	Pediatrics	No	Outcomes; access
Benton et al. (2021) [146]	Spine	No	Outcomes; access
Beck et al. (2021) [147]	Other: general orthopaedics	No	Outcomes; access
Kirchner et al. (2019) [148]	Shoulder and elbow	No	Access
Hartwell (2023) [149]	Shoulder and elbow	No	Outcomes
Garcia et al. (2020) [150]	Shoulder and elbow	No	Outcomes
